# Social Determinants of Self-Reported Health in Vulnerable Populations During a Polycrisis in Lebanon

**DOI:** 10.1001/jamanetworkopen.2025.29733

**Published:** 2025-10-08

**Authors:** Rindala Fayyad, Marie-Elizabeth Ragi, Hala Ghattas, Stephen J. McCall

**Affiliations:** 1 Center for Research on Population and Health, Faculty of Health Sciences, American University of Beirut, Beirut, Lebanon; 2 Department of Health Promotion, Education, and Behavior, University of South Carolina, Columbia

## Abstract

**Importance:**

In the context of a polycrisis, which is characterized by multiple interconnected and mutually reinforcing crises, humanitarian and health needs increase. It is therefore crucial to identify social determinants impacting health and well-being of vulnerable populations to better inform appropriate interventions.

**Objective:**

To assess the association between social determinants and pain frequency, self-rated health, and depression over time among vulnerable populations during a polycrisis in Lebanon.

**Design, Setting, and Participants:**

In this population-based longitudinal cohort study, data were collected in Sin-El-Fil, a suburb of Beirut, Lebanon, in 4 waves: June 28 to October 26, 2022; September 8 to December 22, 2022; January 24 to April 28, 2023; and April 8 to July 5, 2024. Data for this analysis were extracted from a parent study around populations at high risk of COVID-19 infections, morbidity, and mortality and included a sample of Syrian refugees and migrants, pregnant women, older adults, and people of low socioeconomic status. Participants were selected via a multistage stratified sampling design. For this substudy, only individuals residing in neighborhoods with socioeconomic deprivation were considered.

**Exposures:**

Food insecurity, nationality, wealth, educational level, and sex.

**Main Outcomes and Measures:**

The outcomes were pain frequency (days per week), self-rated health (poor or fair, good, or very good or excellent), and depression (measured using the Patient Health Questionnaire–9). Survey-weighted generalized linear regression models were fitted for each outcome-exposure pair, and odds ratios (ORs) and 95% CIs were computed to report mean marginal estimates and time-specific estimates.

**Results:**

The study included 1986 individuals at baseline (mean [SD] age, 44.7 [17.4] years); 1055 (53.1%) were female, 664 (33.4%) were Syrian refugees or migrants, 1025 (51.6%) had no high school degree, and 1451 (73.1%) had food insecurity. The exposure variables were significantly associated with elevated odds of reporting more pain, worse self-rated health, and more depression over time, with mean marginal ORs ranging from 1.03 (95% CI, 1.01-1.06) for worse self-rated health among women compared with men to 1.50 (95% CI, 1.46-1.55) for worse self-rated health among people with vs without food insecurity. The sole exception was for participant sex and depression, between which there was no association (OR, 0.99; 95% CI, 0.97-1.02).

**Conclusions and Relevance:**

In this cohort study, Syrian refugees and migrants, women, and individuals with food insecurity, lower wealth, and less education compared with other residents within the low socioeconomic status areas reported worse physical and mental health challenges, suggesting they carried an additional burden of disadvantage. The findings underscore a need for targeted interventions that focus on reducing health disparities within vulnerable communities, especially in contexts of polycrises.

## Key Points

**Question:**

Are social determinants associated with self-reported health among vulnerable populations facing multiple crises?

**Findings:**

This cohort study following 1986 adults in a Beirut suburb in Lebanon for 2 years found that Syrian refugees and migrants, women, and individuals who had food insecurity, low wealth, and less than a high school education reported more physical pain, worse self-rated health, and more depression than residents without these characteristics.

**Meaning:**

The findings suggest that as global crises continue to affect large populations, interventions that incorporate broader social determinants of health should be implemented to reduce disparities and improve health outcomes within disadvantaged and vulnerable communities.

## Teaser

This cohort study compares self-reported health, pain, and depression among adults with vs without socioeconomic vulnerability factors living in a disadvantaged suburb in Lebanon during a polycrisis.

## Introduction

Since 2019, Lebanon has been facing a severe economic crisis, pushing more than 80% of the population under the poverty level and leaving a destructive impact on the political, educational, security, and health care sectors.^1^ Lebanon has also faced the impacts of the global COVID-19 pandemic and regional conflicts. The economic, health care, and humanitarian crises have created a polycrisis characterized by multiple simultaneous, interconnected, and mutually reinforcing crises, which further increase the risks of adverse health outcomes and exacerbate underlying disparities.^1,2,3,4,5,6,7^ In addition, 2024 recorded the highest number of forcibly displaced people worldwide,^8,9^ and Lebanon continues to host the highest number of refugees per capita, largely emanating from the Syrian war that began in 2011.^10^ The polycrisis has impacted the physical and mental well-being of individuals residing in Lebanon, disproportionately affecting the most vulnerable populations, including refugees, internally displaced people, people in poverty, older adults, women, children, and people with disabilities.^1,4,5^ As state institutions struggle to provide adequate safety nets, the responsibility for meeting needs shifts to humanitarian organizations and local communities.^9,11,12,13^

As the number of people in need of humanitarian assistance in Lebanon and throughout the region increases,^11,12,13^ it is crucial to identify the social determinants that most heavily impact health and well-being in order to better inform interventions. More often than not, individuals in need of humanitarian assistance face high barriers to health care access,^14,15,16^ leading to limited available data on their health status.^17,18,19^ Self-reported health measures, including self-rated health, have been shown to be strongly correlated with objective health^20^ and future risk of mortality.^21,22,23^ It has therefore been argued that self-reported health measures are valid to use as outcomes in research on social determinants of health.^23,24,25,26^ A large body of literature^27,28,29,30,31,32,33,34,35^ has identified social determinants, including sex, race, violence, educational level, household income, employment, and disability, among other factors, to be drivers of poor self-reported physical and mental health. However, a large proportion of these studies were cross-sectional and were conducted in high-income countries (HICs), and limited longitudinal research exists around the predictors of self-reported health in low-or-middle-income countries (LMICs) hosting large refugee populations.

To address this knowledge gap, we conducted a longitudinal telephone survey to assess associations between social determinants and pain frequency, self-rated health, and depression in low socioeconomic status neighborhoods of Sin-El-Fil, a suburb of Beirut, Lebanon, from 2022 to 2024. This is one of the first longitudinal studies, to our knowledge, to investigate these associations across time in both host and refugee populations of an LMIC impacted by a multipronged economic, political, and humanitarian crisis. The findings of this study may inform interventions that can improve the physical and mental health of the most vulnerable populations in contexts of polycrises.

## Methods

### Study Design and Setting

This was a population-based longitudinal cohort study that aimed to investigate the social determinants of self-reported health and well-being among vulnerable populations over time. The study was conducted in Sin-El-Fil, a suburb of Beirut, Lebanon, and the data were collected in 4 waves: wave 1 (June 28 to October 26, 2022), wave 2 (September 8 to December 22, 2022), wave 3 (January 24 to April 28, 2023), and wave 4 (April 8 to July 5, 2024). The study was reviewed and approved by the American University of Beirut’s social and behavioral sciences institutional review board. Oral informed consent was obtained from all participants. The study followed the Strengthening the Reporting of Observational Studies in Epidemiology (STROBE) reporting guideline.

### Study Participants

This study included participants who were selected via a multistage stratified sampling design. The parent study from which the data for this current analysis were extracted focused on vulnerable populations at high risk of COVID-19 infections, morbidity, and mortality.^36^ These individuals included Syrian refugees and migrants, pregnant women, adults aged 60 years or older, and adults of low socioeconomic status.^37,38^ The sampling strategy comprised creating geographic boundaries of neighborhoods in Sin-El-Fil and classifying them based on socioeconomic deprivation. All households within these neighborhoods were listed, enumerated, and surveyed to identify individuals meeting the vulnerability criteria: Syrian refugee or migrant status, pregnancy, age of 60 years or older, and low socioeconomic status. To allow for a balanced and representative cohort, all Syrian individuals and pregnant women were selected due to small sample sizes, while adults aged 60 years or older and adults aged 18 to 60 years from low socioeconomic areas were randomly selected with a proportionate allocation. For this subanalysis, only individuals living in low socioeconomic status areas who participated in the first wave of the study and who had a Lebanese or Syrian nationality were included. After giving oral informed consent, participants were asked to complete a computer-assisted telephone survey and were recontacted at each subsequent wave of the study by trained data collectors to collect follow-up data.

### Data Sources

The survey^39^ was developed using previously validated questions and scales, in collaboration with the Ministry of Public Health, the Sin-El-Fil municipality, and local nongovernmental organizations.^37,38^ The questionnaire was drafted in both English and Arabic, and all data were collected using the SurveyCTO software (Dobility Inc). The data were monitored and quality checked throughout the data collection phase, with 5% of participants called back to check that data were accurate.

### Outcomes

The outcomes were pain frequency, self-rated health, and depression. Pain frequency was measured by asking participants about the number of days they experienced physical pain in a single week, and responses were grouped into 4 categories: never (0 days of pain per week), rarely (1-2 days of pain per week), occasionally (3-4 days of pain per week), and frequently (5 or more days of pain per week). Self-rated health was measured by asking participants to select the term that best described their overall health: *poor*, *fair*, *good*, *very good*, or *excellent*. Responses were then grouped into 3 categories: poor or fair, good, and very good or excellent. Depression was measured using the Patient Health Questionnaire–9 (PHQ-9; score range, 0-27, with higher scores indicating greater depression severity),^40,41^ and the final score was used to group the depression variable into 2 categories: no or minimal depression, or “not depressed” (PHQ-9 score <5), and mild to severe depression, or “depressed” (PHQ-9 score ≥5).

### Exposures

The exposures were food insecurity status, nationality, wealth status, educational level, and sex. Food insecurity was measured using the Arab Family Food Security Scale (AFFSS; score range, 0-7, with higher scores indicating greater food insecurity),^42^ a validated food security tool used in Lebanon. Respondents were asked to respond to a 7-item food insecurity questionnaire, and the final questionnaire score was used to group the food insecurity variable into 2 categories: food insecure (AFFSS score ≥3) and food secure (AFFSS score <3). During initial interviews, participants were asked to report their nationality. Responses were then grouped into 2 categories to create the nationality variable: Syrian refugee or migrant or Lebanese citizen. Wealth status was calculated using a principal-component analysis of 17 different types of assets^37^ and was grouped into 2 categories: low wealth (first tertile of wealth score) and middle or high wealth (second and third tertiles of wealth score). Educational level was classified as having less than a high school degree vs having a high school degree or higher. Sex included females and males.

### Confounders

**Figure 1.**
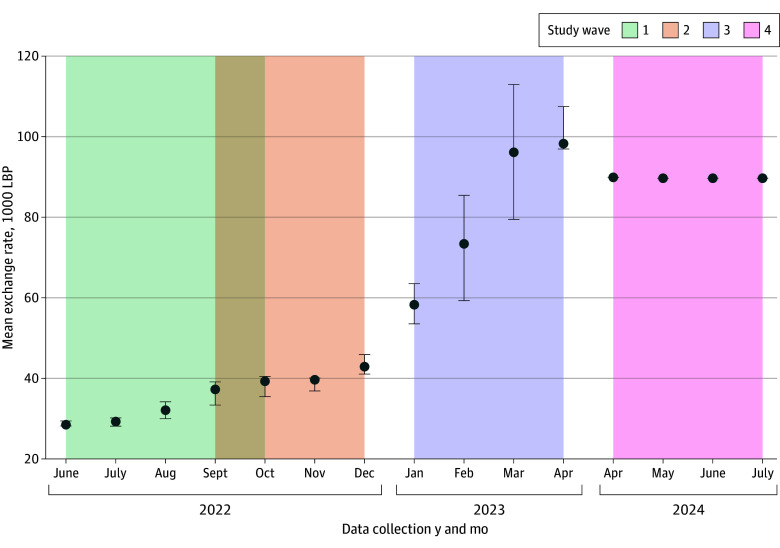
Changes in the US Dollar–Lebanese Pound (LBP) Exchange Rate During the Study Period Data points represent means and whiskers, ranges.

Confounders were identified and adjusted for using directed acyclic graphs (eFigure 1 in Supplement 1). Variables confounding the association between food insecurity and each of the outcomes included age, sex, nationality, employment status, crowding index (defined as the number of people per room in the household), wealth status, and the US dollar (USD) to Lebanese pound (LBP) exchange rate to adjust for major currency fluctuations that occurred during the conduct of the study (Figure 1) (each respondent was assigned the daily currency exchange rate corresponding to the date their survey was conducted). Variables confounding the association between nationality and the outcomes included age, sex, and USD-LBP exchange rate. Variables confounding the association between wealth and the outcomes included age, sex, nationality, employment status, and USD-LBP exchange rate. Variables confounding the association between educational level and the outcomes included age, sex, nationality, and USD-LBP exchange rate. Variables confounding the association between sex and the outcomes included age and USD-LBP exchange rate.

### Statistical Analysis

Study participants were followed up until the final wave, even in cases of nonresponse to follow-up in intermediate waves. Descriptive analyses were used to describe demographics, exposures, and outcomes and to investigate the unadjusted associations between the variables at each wave and month of the study.

To study the association between each exposure and outcome of interest, we fitted survey-weighted generalized linear regression models using the survey package in R, version 4.4-2 (R Project for Statistical Computing),^43^ which took into account the study’s complex survey design and included design-based SEs and inverse-probability weights to account for nonresponse bias (further details are included in the eMethods in Supplement 1). Separate models were fitted for each outcome-exposure pair, and each model included the main term for the exposure, the main term for time (treated as a categorical variable for study wave), interaction terms between the exposure and time variables, and adjustment terms for confounders. To assess the association of the exposure with the outcome, odds ratios (ORs) and 95% CIs were computed at each wave of the study while holding all other variables constant. Mean marginal estimates were also computed to assess the overall mean OR between the exposure and outcome across all waves.

Variables had at most 5.3% missing data (eTable 1 in Supplement 1), and values were imputed using predictive mean matching, a semiparametric multiple imputation approach. Data cleaning, extraction, and manipulation were conducted using Stata, version 18 (StataCorp LLC), and R, version 4.4-2, and all statistical analyses were conducted using R, version 4.4-2.

## Results

**Figure 2.**
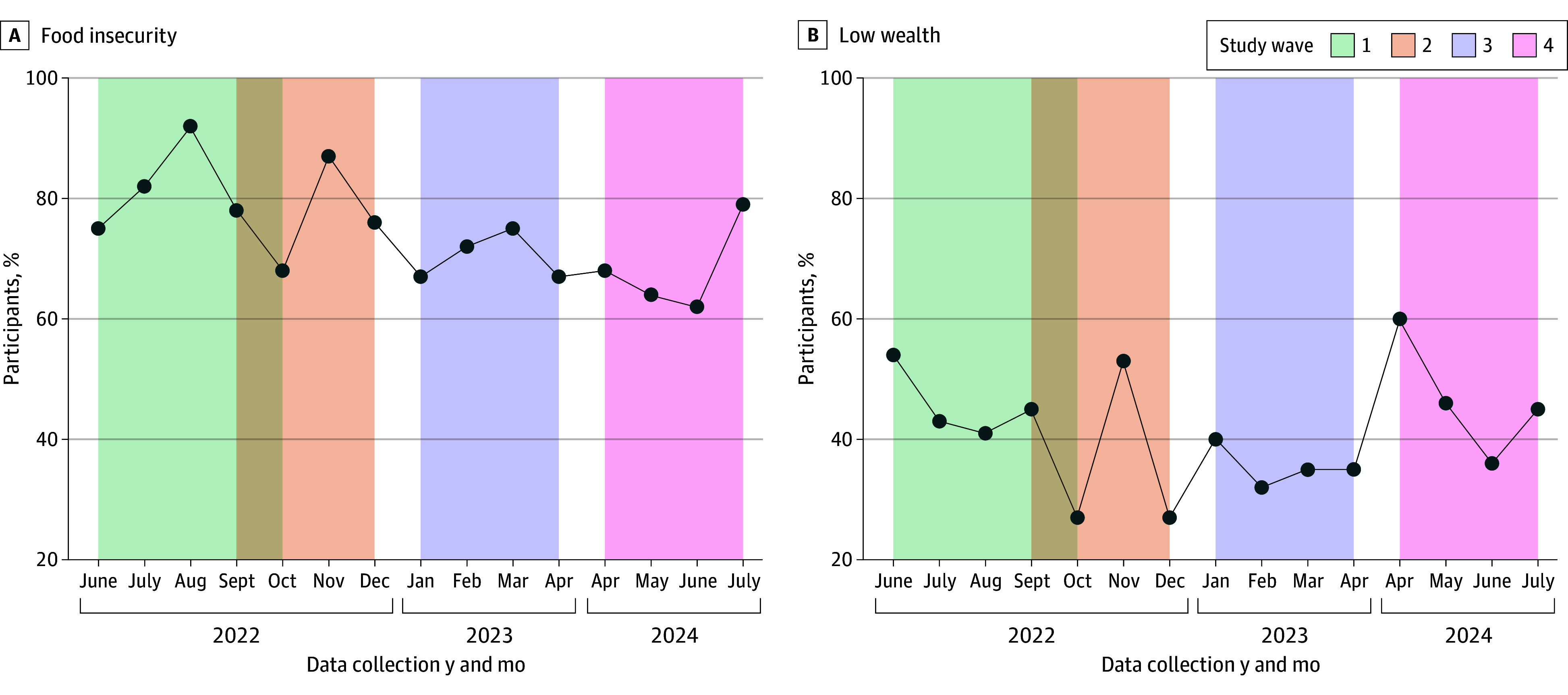
Changes in Prevalence of Food Insecurity and Low Wealth Status Among Survey Participants Across Time

**Figure 3.**
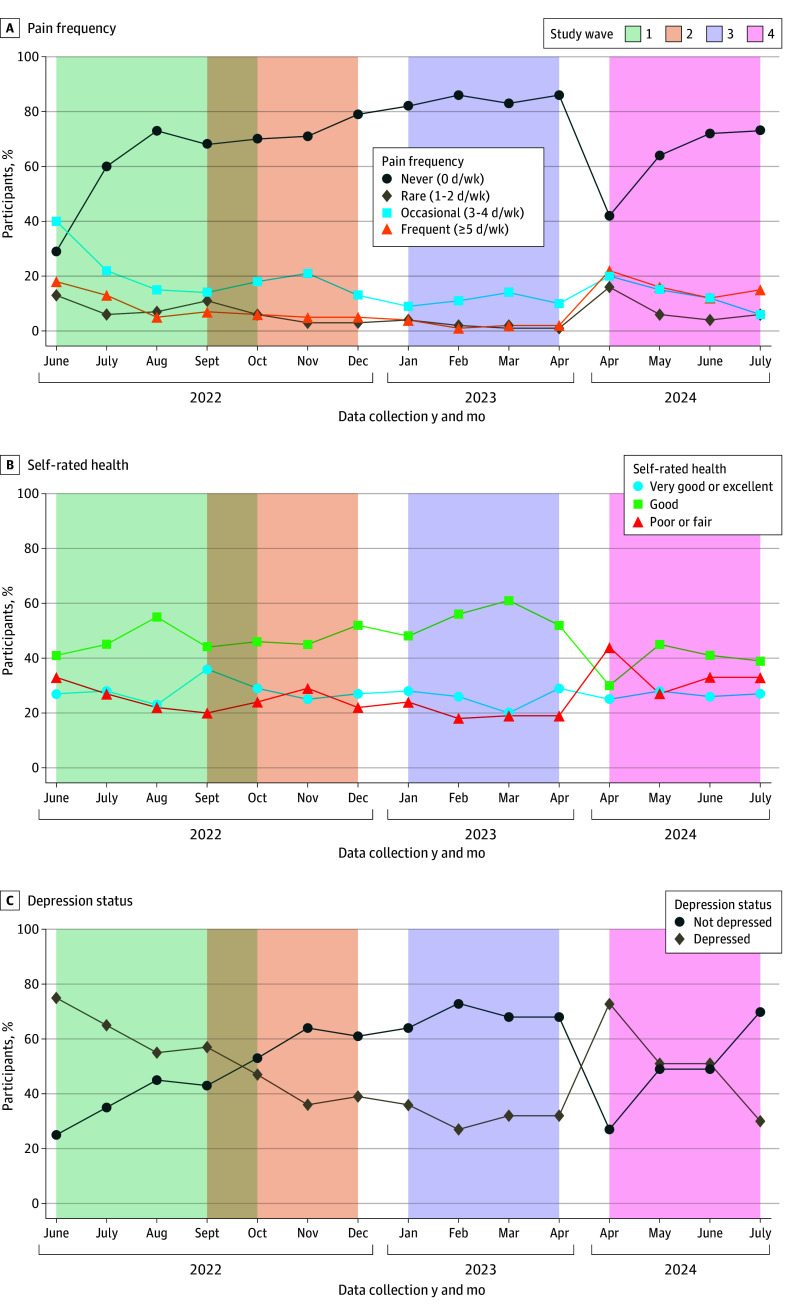
Changes in Prevalence of Outcomes Among Survey Participants Across Time

From the study population residing in low socioeconomic areas (n = 2045), this subanalysis included 1986 individuals at baseline, out of which 1675 (84.3%) remained at wave 2, 1594 (80.3%) at wave 3, and 951 (47.9%) at wave 4. At baseline, the mean (SD) age was 44.7 (17.4) years; 1055 participants (53.1%) were female, 931 (46.9%) were male, 664 (33.4%) were Syrian refugees or migrants, 1025 (51.6%) had less than a high school degree, and 1451 (73.1%) had food insecurity (Table 1). Regarding the outcome variables at baseline, 700 participants (35.3%) reported experiencing pain at least once per week; 1167 (58.8%) had depression; and 445 (22.4%), 892 (44.9%), and 649 (32.7%) described their overall health as poor or fair, good, and very good or excellent, respectively (Table 1). Changes in prevalence of exposure and outcome variables among study participants across time can be found in Figure 2 and Figure 3, and changes in the percentage of individuals experiencing simultaneous exposures at each wave of the study can be found in eFigure 2 in Supplement 1.

**Table 1.** Characteristics of the Study Participants at Each Study Wave

Characteristic	Participants^a^			
	Wave 1 (n = 1986)	Wave 2 (n = 1675)	Wave 3 (n = 1594)	Wave 4 (n = 951)
Age, mean (SD), y	44.7 (17.4)	44.6 (17.3)	43.9 (17.1)	46.0 (16.5)
Sex				
Female	1055 (53.1)	868 (51.8)	852 (53.5)	502 (52.8)
Male	931 (46.9)	807 (48.2)	742 (46.5)	449 (47.2)
Nationality				
Lebanese	1322 (66.6)	1054 (62.9)	990 (62.1)	559 (58.8)
Syrian refugee or migrant	664 (33.4)	621 (37.1)	604 (37.9)	392 (41.2)
Marital status				
Single or engaged	548 (27.6)	444 (26.5)	426 (26.7)	212 (22.3)
Married	1259 (63.4)	1085 (64.8)	1027 (64.4)	661 (69.5)
Separated or widowed	179 (9.0)	146 (8.7)	141 (8.8)	78 (8.2)
Educational level				
≥High school degree	961 (48.4)	801 (47.8)	761 (47.7)	473 (49.7)
<High school degree	1025 (51.6)	874 (52.2)	833 (52.3)	478 (50.3)
Employment status				
Employed	847 (42.6)	700 (41.8)	637 (40.0)	345 (36.3)
Unemployed	1139 (57.4)	975 (58.2)	957 (60.0)	606 (63.7)
Crowding index				
<2 People per room	976 (49.1)	777 (46.4)	738 (46.3)	394 (41.4)
2-3 People per room	620 (31.2)	536 (32.0)	507 (31.8)	307 (32.3)
≥3 People per room	390 (19.6)	362 (21.6)	349 (21.9)	250 (26.3)
Wealth status				
Middle or highest tertile	1308 (65.9)	1045 (62.4)	1033 (64.8)	507 (53.3)
Lowest tertile	678 (34.1)	630 (37.6)	561 (35.2)	444 (46.7)
Food insecurity status				
Insecure	1451 (73.1)	1382 (82.5)	1122 (70.4)	619 (65.1)
Secure	535 (26.9)	293 (17.5)	472 (29.6)	332 (34.9)
Self-rated health				
Very good or excellent	649 (32.7)	415 (24.8)	395 (24.8)	254 (26.7)
Good	892 (44.9)	849 (50.7)	886 (55.6)	381 (40.1)
Poor or fair	445 (22.4)	411 (24.5)	313 (19.6)	316 (33.2)
Pain frequency				
Never (0 d/wk)	1286 (64.7)	1246 (74.4)	1338 (83.9)	576 (60.6)
Rare (1-2 d/wk)	202 (10.2)	52 (3.1)	31 (1.9)	74 (7.8)
Occasional (3-4 d/wk)	304 (15.3)	306 (18.3)	192 (12.0)	144 (15.1)
Frequent (≥5 d/wk)	194 (9.8)	71 (4.2)	33 (2.1)	157 (16.5)
Depression status				
None or minimal	819 (41.2)	982 (58.6)	1088 (68.3)	421 (44.3)
Mild to severe	1167 (58.8)	693 (41.4)	506 (31.7)	530 (55.7)
USD-LBP exchange rate, median (range)	35 500 (28 200-40 400)	39 900 (35 300-45 900)	89 500 (53 400-113 000)	89 700 (89 700-89 700)

Abbreviations: LBP, Lebanese pound; USD, US dollar.

^a^ Data are presented as number (percentage) of participants unless otherwise indicated.

Food insecurity, nationality, low wealth status, low educational level, and sex were all significantly associated with elevated odds of each of the outcomes of interest except for sex and depression, between which there was no association. People with food insecurity had higher odds of increased pain frequency (OR, 1.09; 95% CI, 1.06-1.12), worse self-rated health (OR, 1.50; 95% CI, 1.46-1.55), and depression (OR, 1.18; 95% CI, 1.15-1.22) compared with people with food security, on average (Table 2).

**Table 2.** Marginal Estimates of the ORs and 95% CIs of Experiencing Increased Pain Frequency, Worse Self-Rated Health, and Depression for Each Exposure of Interest Across Time

Exposure	Outcome, OR (95% CI)		
	Increased pain frequency	Worse self-rated health	Depression
Food insecurity status			
Secure	1 [Reference]	1 [Reference]	1 [Reference]
Insecure	1.09 (1.06-1.12)	1.50 (1.46-1.55)	1.18 (1.15-1.22)
Nationality			
Lebanese citizen	1 [Reference]	1 [Reference]	1 [Reference]
Syrian refugee or migrant	1.11 (1.09-1.14)	1.12 (1.09-1.15)	1.14 (1.11-1.18)
Wealth status			
Middle or highest tertile	1 [Reference]	1 [Reference]	1 [Reference]
Lowest tertile	1.07 (1.04-1.11)	1.11 (1.07-1.14)	1.11 (1.07-1.15)
Educational level			
≥High school degree	1 [Reference]	1 [Reference]	1 [Reference]
<High school degree	1.06 (1.04-1.09)	1.08 (1.05-1.11)	1.06 (1.03-1.09)
Sex			
Female	1.06 (1.04-1.09)	1.03 (1.01-1.06)	0.99 (0.97-1.02)
Male	1 [Reference]	1 [Reference]	1 [Reference]

Abbreviation: OR, odds ratio.

Syrian refugees and migrants had higher odds of increased pain frequency (OR, 1.11; 95% CI, 1.09-1.14), worse self-rated health (OR, 1.12; 95% CI, 1.09-1.15), and depression (OR, 1.14; 95% CI, 1.11-1.18) compared with Lebanese citizens, on average. People with the lowest wealth status had higher odds of increased pain frequency (OR, 1.07; 95% CI, 1.04-1.11), worse self-rated health (OR, 1.11; 95% CI, 1.07-1.14), and depression (OR, 1.11; 95% CI, 1.07-1.15) compared with those with middle or high wealth status, on average (Table 2).

People without a high school degree had higher odds of increased pain frequency (OR, 1.06; 95% CI, 1.04-1.09), worse self-rated health (OR, 1.08; 95% CI, 1.05-1.11), and depression (OR, 1.06; 95% CI, 1.03-1.09) compared with those with a high school degree or higher, on average. In addition, women had higher odds of increased pain frequency (OR, 1.06; 95% CI, 1.04-1.09) and worse self-rated health (OR, 1.03; 95% CI, 1.01-1.06) compared with men, on average (Table 2). All ORs were estimated while holding other variables constant. Time-specific ORs varied from one follow-up period to the other, and at some of the waves, the ORs were significantly higher compared with previous or subsequent waves; separate adjusted OR estimates and 95% CIs for each wave of the study can be found in eTable 2 in Supplement 1.

## Discussion

In this longitudinal cohort study of 1986 individuals from vulnerable populations, food insecurity, Syrian refugee and migrant status, low wealth, lack of a high school degree, and being of female sex were associated with significantly increased odds of respondents reporting increased pain frequency, worse self-rated health, and (except for female sex) depression across time. Many cross-sectional,^29,44,45,46,47,48,49,50,51,52,53,54^ review,^30,33,34,35,55,56,57,58,59,60^ and longitudinal^61,62,63,64,65,66^ studies have previously identified associations of social factors with objective health outcomes and self-reported health outcomes; however, most of the existing research was set in HICs. Supporting the current study’s findings, the literature consistently demonstrated that social determinants are significantly associated with health and well-being and are key drivers of health disparities, with lower socioeconomic groups experiencing worse health outcomes regardless of broader health care system differences.^31,45,49,53,54,57,61^ The literature has also shown, in accordance with our findings, that food insecurity, low educational level, economic hardship, and limited access to quality health care are associated with poorer mental and physical health.^14,16,44,46,47,50,67^ Race, refugee, and asylum-seeking status were found to further exacerbate these disparities, but most of the current literature on refugee status and health focused on displaced individuals who have resettled in HICs.^13,51,52,56,57,60,65,68,69,70^

Therefore, this study is an important addition to the literature because there are limited studies investigating the social determinants of self-reported health of refugees and host populations of LMICs living side by side in the same community. This longitudinal study focused on populations who were either refugees and migrants or citizens of an LMIC facing a polycrisis. Additionally, in the context of this study, Syrian refugee and migrant status was treated as an independent social determinant of health, separate from other factors such as wealth, educational level, or socioeconomic status. The findings presented herein on the association between nationality and self-reported health suggest that refugee and migrant status remains a critical determinant of health, especially given the protracted nature of displacement in Lebanon, where many refugees have lived in long-term unstable conditions that have continuously affected their well-being.^5,71,72,73^

The findings of this study have important programmatic and policy implications. The results suggest that vulnerable populations living in low socioeconomic status environments carry an additional burden of disadvantage compared with other residents in similar socioeconomic conditions. While poverty itself is a major determinant of health, factors such as food insecurity, refugee status, low educational level, and female sex may further worsen health challenges, leaving individuals in the most precarious situations with the worst health outcomes. Additionally, while refugee status, educational level, and sex are relatively stable characteristics and are generally fixed over time, wealth and food insecurity may not be. The potential variability of these factors places social protection as a key intervention during times of crisis to prevent the exacerbation of underlying vulnerabilities, particularly for individuals at high risk of becoming food insecure or losing wealth. In addition, given that the percentage of individuals with risk factors for poor health outcomes in Lebanon, throughout the region, and in conflict zones has seen a dramatic increase in recent years,^8,11^ policy makers and humanitarian organizations should ensure the inclusion of social determinants of health in the design of targeted interventions, programs, and emergency preparedness and response in order to prevent further exacerbation of health inequalities. Examples of such interventions may include expanding food assistance programs in areas with high food insecurity, offering cash assistance, and providing winterization assistance programs.

This study contributes to the growing literature on social determinants of health and self-reported health. Future similar studies should be conducted using larger samples of people from LMICs, and estimation of changes in exposure effects across different time points should also be investigated.

### Strengths and Limitations

A strength of this study lies in its design and setting. Study participants were followed throughout a critical 2-year period in Lebanon, which saw striking changes in inflation rate, value of goods and services, health care, political unrest, and conflict. Even after adjusting for contextual variables, including fluctuations in the local currency value, the associations between the exposure and outcome variables persisted. In addition, at some of the waves, the ORs were significantly higher compared with previous or subsequent waves (eTable 2 in Supplement 1). This highlights the importance of longitudinal research and of incorporating sociopolitical events into longitudinal research models, as individual data collected at 2 different time points can yield substantially different results even if the periods are close together when significant social, environmental, or political events occur during the intervening time.

Several limitations of our study should also be noted. Although the data were frequently monitored in parallel with quality assurance, there was still a possibility for minor variable misclassification due to the self-reported nature of the data. In addition, while we adjusted for a range of confounders, there could still be additional unmeasured confounders for which we did not account. Also, due to the limited and decreasing amount of data at each wave of the study, some of the time-specific estimates may have been unstable. However, these limitations did not hinder the study’s objective of identifying associations between social determinants of health and pain frequency, self-rated health, and depression.

## Conclusions

This cohort study investigated associations across time between social factors and physical and mental health among vulnerable populations living in Lebanon, a country facing overlapping economic, political, and humanitarian crises. The findings revealed that food insecurity, nationality, wealth status, educational level, and sex were significantly associated with pain frequency, self-rated health, and depression. Policy makers and humanitarian actors can use the evidence presented in this study to improve physical and mental well-being by using a social determinants of health lens, especially among the most vulnerable populations living in crisis-dominated areas.

## Notes

Supplement 1. **eMethods.** Nonresponse Inverse-Probability Weights (IPWs) **eFigure 1.** Directed Acyclic Graph (DAG) for the Relationship Between the Exposure and Outcome Variables **eFigure 2.** Percentage of Individuals Experiencing Simultaneous Exposures at Each Wave of the Study **eTable 1.** Distribution of the Study Variables Across All Waves, Including Percentage of Missing Data **eTable 2.** Estimates of the Odds Ratios and 95% CIs of Experiencing Increased Pain Frequency, Worse Self-Rated Health, and Depression for Each Exposure of Interest at Each Wave of the Study

Supplement 2.
Community Action for Equity in Pandemic Preparedness and Control (CAEP) Study Group

Supplement 3.
Data Sharing Statement
