# Supplementary material for: Social Determinants of Self-Reported Health in Vulnerable Populations During a Polycrisis in Lebanon
Source: JAMA Netw Open. 2025 Oct 8;8(10):e2529733. doi: 10.1001/jamanetworkopen.2025.29733 (PMC12509029; doi:10.1001/jamanetworkopen.2025.29733)
Supplement: Supplement 1. — eMethods. Nonresponse Inverse-Probability Weights (IPWs) eFigure 1. Directed Acyclic Graph (DAG) for the Relationship Between the Exposure and Outcome Variables eFigure 2. Percentage of Individuals Experiencing Simultaneous Exposures at Each Wave of the Study eTable 1. Distribution of the Study Variables Across All Waves, Including Percentage of Missing Data eTable 2. Estimates of the Odds Ratios and 95% CIs of Experiencing Increased Pain Frequency, Worse Self-Rated Health, and Depression for Each Exposure of Interest at Each Wave of the Study [file jamanetwopen-e2529733-s001.pdf]

Supplementary Online Content

Fayyad R, Ragi ME, Ghattas H, McCall SJ; for the Community Action for Equity in Pandemic Preparedness and Control (CAEP) Study Group. Social determinants of self-reported health in vulnerable populations during a polycrisis in Lebanon. *JAMA Netw Open*. 2025;8(10):e2529733. doi:10.1001/jamanetworkopen.2025.29733

eMethods. Nonresponse Inverse-Probability Weights (IPWs)

eFigure 1. Directed Acyclic Graph (DAG) for the Relationship Between the Exposure and Outcome Variables

eFigure 2. Percentage of Individuals Experiencing Simultaneous Exposures at Each Wave of the Study

eTable 1. Distribution of the Study Variables Across All Waves, Including Percentage of Missing Data

eTable 2. Estimates of the Odds Ratios and 95% CIs of Experiencing Increased Pain Frequency, Worse Self-Rated Health, and Depression for Each Exposure of Interest at Each Wave of the Study

This supplementary material has been provided by the authors to give readers additional information about their work.

© 2025 Fayyad R et al. *JAMA Network Open*.

eMethods: Nonresponse Inverse-Probability Weights (IPWs)

In this section, we provide details on how we mitigated non-response bias at each wave of the study using inverse probability weights (IPWs), which we then implemented into the regression models to evaluate the marginal associations between each exposure and outcome of interest.

1- Base weights

For each individual, i, the base weight (i.e., selection weight), $w_{0,ij}$, was calculated as the inverse of their selection probability:

$$w_{0,ij} = \frac{1}{\pi_{ij}} = \frac{1}{\frac{n_j}{N_j}} = \frac{N_j}{n_j}$$

where n_j is the total number of individuals contacted in sampling grid j, and N_j is the total number of persons listed in sampling grid j.

2- Non-response at wave 1

A response propensity model was fitted to account for non-response during the first wave of data collection. The model was of the form:

$$\begin{aligned}\text{logit}(\text{E}[\text{I}(\text{Responded in wave 1})] \\ &= \beta_0 + \beta_1 \cdot \text{Sampling grid number} + \beta_2 \cdot \text{SES of grid} + \beta_3 \cdot \text{Household size} \\ &+ \beta_4 \cdot \text{I}(\text{Household with older adult}) + \beta_5 \cdot \text{I}(\text{Household with pregnant women}) \\ &+ \beta_6 \cdot \text{I}(\text{Household with Syrian refugees})\end{aligned}$$

where I = indicator variable & $\beta_{p|p = \{0,\ldots,6\}}$ are the model coefficients.

Using the above model, propensity scores (i.e., probability of responding to wave 1 for each individual) were estimated, grouped into deciles, and the mean propensity score for each decile, $a_{d|d=\{1,\ldots,10\}}$, was calculated. The non-response-adjusted IPW at wave 1, $w_{1,ijd}$, for each individual respondent within decile d was equal to:

$$w_{1,ijd} = w_{0,ij} \cdot \frac{1}{a_d}$$

This is referred to as the propensity stratification method.[1]

Additionally, to minimize the impact of extreme weights, they were trimmed at the 90th percentile, as values above this threshold were disproportionately large and risked inflating the variance of model estimates. The table below presents a summary and comparison of the weights before and after trimming:

	Minimum	1st quantile	Median	Mean	3rd quantile	Maximum	Unequal weighting effect
Before trimming	1.29	1.90	3.31	3.39	4.42	9.48	1.21
After trimming	1.29	1.90	3.31	3.31	4.42	5.58	1.18

3- Non-response at wave 2

At wave 2, we recontacted everyone who responded in wave 1 of the study. In a similar approach to wave 1, a response propensity model was fitted to account for non-response while adjusting for variables possibly influencing one's ability or willingness to respond in wave 2, after having responded in wave 1. Variables included in the model

© 2025 Fayyad R et al. *JAMA Network Open*.

below were extracted from the data collected in wave 1:

$$\text{logit}(\text{E}[\text{I}(\text{Responded in wave 2})]) = \alpha_0 + \alpha_1 \cdot \text{Age} + \alpha_2 \cdot \text{Sex} + \alpha_3 \cdot \text{Nationality} + \alpha_4 \cdot \text{Employment status} + \alpha_5 \cdot \text{Self rated health} + \alpha_6 \cdot \text{I(Presence of chronic condition)} + \alpha_7 \cdot \text{I(Presence of disability)} + \alpha_8 \cdot \text{I(Required hospitalization)} + \alpha_9 \cdot \text{I(Water insecurity status)} + \alpha_{10} \cdot \text{I(Food insecurity status)} + \alpha_{11} \cdot \text{Wealth} + \alpha_{12} \cdot \text{Depression level}$$

where I = indicator variable & $\alpha_{p|p=\{0,\dots,12\}}$ are the model coefficients.

Using the above model, propensity scores were estimated, grouped into deciles, and the mean propensity score for each decile, $b_{d|d=\{1,\dots,10\}}$, was calculated. The non-response IPW at wave 2, $w_{2,ijd}$, for each individual respondent within decile d was equal to:

$$w_{2,ijd} = w_{1,ijd} \cdot \frac{1}{b_d}$$

To be consistent with the approach implemented in wave 1, weights were trimmed at the 90th percentile. The table below presents a summary and comparison of the weights before and after trimming:

	Minimum	1st quantile	Median	Mean	3rd quantile	Maximum	Unequal weighting effect
Before trimming	1.34	1.62	3.95	5.54	5.01	101.73	2.81
After trimming	1.34	1.62	3.95	4.45	5.01	12.08	1.51

4- Non-response at wave 3

At wave 3, we recontacted everyone who responded in wave 1 of the study, regardless of whether they responded in wave 2 or not. A response propensity model was fitted to account for non-response while adjusting for variables possibly influencing one's ability or willingness to respond in wave 3, after having responded in at least one of the previous 2 waves. Variables included in the model below were extracted from the data collected in the most recently observed wave for each individual. For instance, if individual A responded in waves 1 and 2, then data for that individual were extracted from wave 2; and if individual B responded in wave 1 but not wave 2, then data for that individual were extracted from wave 1. Using that information, we fit the following response propensity model:

$$\text{logit}(\text{E}[\text{I}(\text{Responded in wave 3})]) = \gamma_0 + \gamma_1 \cdot \text{Age} + \gamma_2 \cdot \text{Sex} + \gamma_3 \cdot \text{Nationality} + \gamma_4 \cdot \text{Employment status} + \gamma_5 \cdot \text{Self rated health} + \gamma_6 \cdot \text{I(Presence of chronic condition)} + \gamma_7 \cdot \text{I(Presence of disability)} + \gamma_8 \cdot \text{I(Required hospitalization)} + \gamma_9 \cdot \text{I(Water insecurity status)} + \gamma_{10} \cdot \text{I(Food insecurity status)} + \gamma_{11} \cdot \text{Wealth} + \gamma_{12} \cdot \text{Depression level}$$

where I = indicator variable & $\gamma_{p|p=\{0,\dots,12\}}$ are the model coefficients.

Using the above model, propensity scores were estimated, grouped into deciles, and the mean propensity score for each decile, $c_{d|d=\{1,\dots,10\}}$, was calculated. The non-response IPW at wave 3, $w_{3,ijd}$, for each individual respondent within decile d was equal to:

$$w_{3,ijd} = w_{x,ijd} \cdot \frac{1}{c_d}$$

Where $w_{x,ijd} = w_{2,ijd}$ for individuals who responded in wave 2 and $w_{x,ijd} = w_{1,ijd}$ otherwise.

To be consistent with the approach implemented in waves 1 and 2, weights were trimmed at the 90th percentile. The

© 2025 Fayyad R et al. *JAMA Network Open*.

table below presents a summary and comparison of the weights before and after trimming:

	Minimum	1st quantile	Median	Mean	3rd quantile	Maximum	Unequal weighting effect
Before trimming	1.39	1.96	4.83	6.32	6.42	108.22	2.43
After trimming	1.39	1.96	4.83	5.09	6.42	11.31	1.37

5- Non-response at wave 4

At wave 4, we recontacted everyone who responded in wave 1 of the study, regardless of whether they responded in wave 3 or not. A response propensity model was fitted to account for the non-response bias while adjusting for variables possibly influencing one's ability or willingness to respond in wave 4, after having responded in at least one of the previous 3 waves. Similar to the approach implemented in wave 3, variables included in the model below were extracted from the data collected in the most recently observed wave for each individual:

$$\begin{aligned}\text{logit}(\text{E}[\text{I}(\text{Responded in wave 4})]) &= \theta_0 + \theta_1\cdot\text{Age} + \theta_2\cdot\text{Sex} + \theta_3\cdot\text{Nationality} + \theta_4\cdot\text{Employment status} + \theta_5\cdot\text{Self rated health} \\ &+ \theta_6\cdot\text{I}(\text{Presence of chronic condition}) + \theta_7\cdot\text{I}(\text{Presence of disability}) \\ &+ \theta_8\cdot\text{I}(\text{Required hospitalization}) + \theta_9\cdot\text{I}(\text{Water insecurity status}) \\ &+ \theta_{10}\cdot\text{I}(\text{Food insecurity status}) + \theta_{11}\cdot\text{Wealth} + \theta_{12}\cdot\text{Depression level}\end{aligned}$$

where I = indicator variable & $\theta_{p|p = \{0,\ldots,12\}}$ are the model coefficients.

Using the above model, propensity scores were estimated, grouped into deciles, and the mean propensity score for each decile, $e_{d|d=\{1,\ldots,10\}}$, was calculated. The non-response IPW at wave 4, $w_{4,ijd}$, for each individual respondent within decile d was equal to:

$$w_{4,ijd} = w_{y,ijd} \cdot \frac{1}{e_d}$$

Where $w_{y,ijd} = w_{3,ijd}$ for individuals who responded in wave 3 and $w_{y,ijd} = w_{x,ijd}$ otherwise.

To be consistent with the approach implemented in the previous three waves, weights were trimmed at the 90th percentile. The table below presents a summary and comparison of the weights before and after trimming:

	Minimum	1st quantile	Median	Mean	3rd quantile	Maximum	Unequal weighting effect
Before trimming	1.74	3.77	6.85	7.93	10.67	45.41	1.42
After trimming	1.74	3.77	6.85	7.45	10.67	14.37	1.29

$\{w_1, w_2, w_3, w_4\}$ are the final non-response-adjusted inverse-probability weights that were implemented in the models for waves 1, 2, 3, and 4, respectively.

© 2025 Fayyad R et al. *JAMA Network Open*.

eFigure 1: Directed Acyclic Graph (DAG) for the Relationship Between the Exposure and Outcome Variables

The graph below allowed us to identify which set of variables should be adjusted for in our models in order to minimize confounding bias. Each arrow (representing a suspected causal association) was justified through literature reviews. The final DAG was agreed upon after consulting with all team members, and discussing the pathways and underlying assumptions behind each element of the DAG.

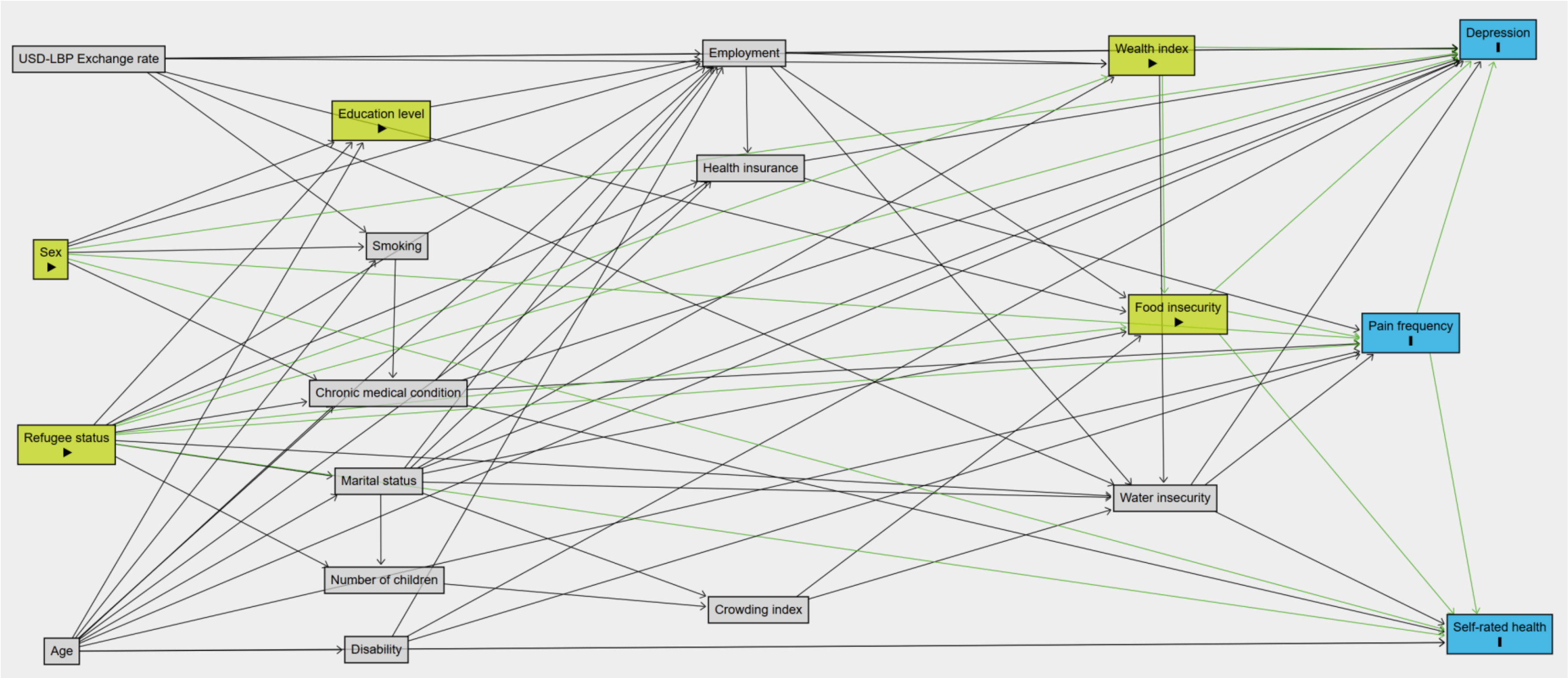

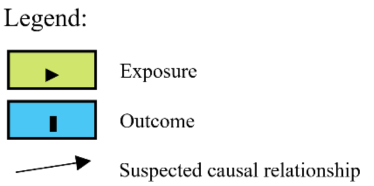

© 2025 Fayyad R et al. *JAMA Network Open*.

eFigure 2: Percentage of Individuals Experiencing Simultaneous Exposures at Each Wave of the Study

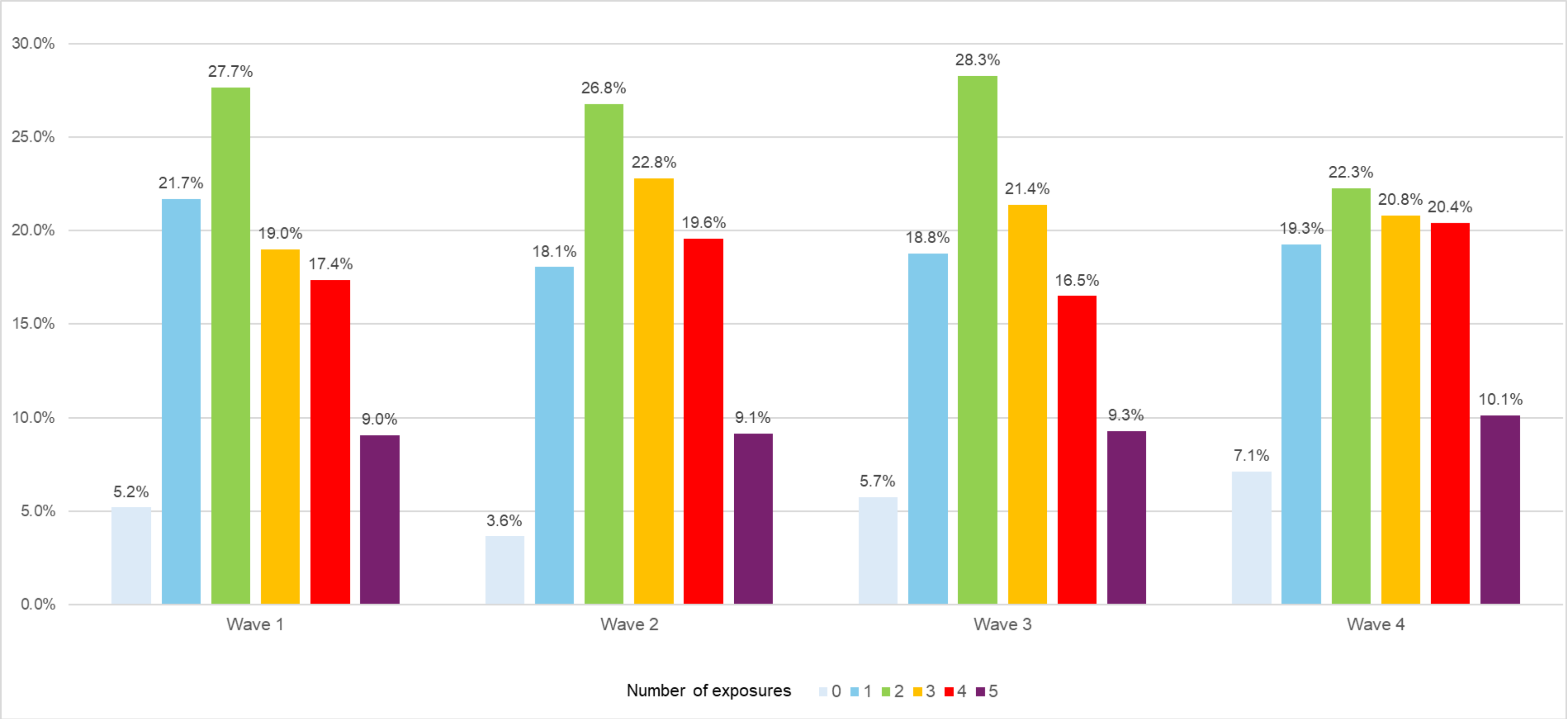

© 2025 Fayyad R et al. *JAMA Network Open*.

eTable 1: Distribution of the Study Variables Across All Waves, Including Percentage of Missing Data

Characteristic	All observations (n = 6206)
**Age**	
Mean (SD), y	44.6 (17.1)
Missing	13 (0.2%)
**Sex**	
Female	3277 (52.8%)
Male	2929 (47.2%)
Missing	0 (0%)
**Nationality**	
Lebanese	3925 (63.2%)
Syrian refugee or migrant	2281 (36.8%)
Missing	0 (0%)
**Marital status**	
Single or engaged	1630 (26.3%)
Married	4032 (65.0%)
Separated or widowed	544 (8.8%)
Missing	0 (0%)
**Educational level**	
≥High school degree	2843 (45.8%)
<High school degree	3036 (48.9%)
Missing	327 (5.3%)
**Employment status**	
Employed	2527 (40.7%)
Unemployed	3665 (59.1%)
Missing	14 (0.2%)
**Crowding index**	
<2 People per room	2856 (46.0%)
2-3 People per room	1904 (30.7%)
≥3 People per room	1199 (19.3%)
Missing	247 (4.0%)
**Wealth status**	
Middle or highest tertile	3865 (62.3%)
Lowest tertile	2341 (37.7%)
Missing	0 (0%)
**Food insecurity status**	
Insecure	4377 (70.5%)
Secure	1565 (25.2%)
Missing	264 (4.3%)
**Self-rated health**	
Very good or excellent	1713 (27.6%)
Good	3008 (48.5%)
Poor or fair	1485 (23.9%)
Missing	0 (0%)
**Pain frequency**	
Never (0 d/wk)	4446 (71.6%)
Rare (1-2 d/wk)	359 (5.8%)
Occasional (3-4 d/wk)	946 (15.2%)
Frequent (≥5 d/wk)	455 (7.3%)
Missing	0 (0%)

© 2025 Fayyad R et al. *JAMA Network Open*.

Characteristic	All observations (n = 6206)
**Depression status**	
None or minimal	3290 (53.0%)
Mild to severe	2840 (45.8%)
Missing	76 (1.2%)
**USD-LBP exchange rate**	
Median (range)	41 100 (28 200-113 000)
Missing	0 (0.0%)

© 2025 Fayyad R et al. *JAMA Network Open*.

eTable 2: Estimates of the Odds Ratios and 95% CIs of Experiencing Increased Pain Frequency, Worse Self-Rated Health, and Depression For Each Exposure of Interest at Each Wave of the Study

Exposure	Wave 1	Wave 2	Wave 3	Wave 4
	**Increased pain frequency**			
Food insecure vs food secure	0.98 (0.77-1.25)	1.99 (1.36-2.89)	3.49 (2.42-5.03)	2.03 (1.42-2.91)
Syrian refugee or migrant vs Lebanese citizen	2.27 (1.80-2.85)	2.12 (1.62-2.76)	0.76 (0.54-1.07)	2.36 (1.72-3.24)
Low wealth vs middle or high wealth	1.28 (0.99-1.65)	1.69 (1.28-2.24)	1.38 (0.99-1.92)	1.63 (1.15-2.30)
<High school degree vs ≥high school degree	1.36 (1.10-1.68)	1.25 (0.97-1.60)	1.57 (1.16-2.15)	1.74 (1.25-2.40)
Female vs Male	1.51 (1.22-1.86)	1.38 (1.08-1.77)	1.30 (0.96-1.76)	1.43 (1.03-1.99)
	**Worse self-rated health**			
Food insecure vs food secure	2.44 (1.95-3.05)	9.28 (6.77-12.73)	41.86 (29.09-60.22)	6.62 (4.60-9.51)
Syrian refugee or migrant vs Lebanese citizen	1.50 (1.21-1.87)	1.81 (1.39-2.34)	3.22 (2.36-4.40)	1.41 (1.03-1.94)
Low wealth vs middle or high wealth	1.06 (0.83-1.35)	1.17 (0.87-1.56)	4.78 (3.16-7.21)	2.91 (2.03-4.19)
<High school degree vs ≥high school degree	1.89 (1.54-2.33)	1.37 (1.07-1.75)	1.00 (0.77-1.30)	1.81 (1.29-2.53)
Female vs Male	1.24 (1.02-1.52)	1.16 (0.91-1.49)	1.16 (0.90-1.51)	1.09 (0.78-1.52)
	**Depression**			
Food insecure vs food secure	2.45 (1.97-3.05)	3.84 (2.70-5.45)	3.21 (2.33-4.42)	0.43 (0.31-0.59)
Syrian refugee or migrant vs Lebanese citizen	1.89 (1.53-2.34)	1.66 (1.33-2.08)	2.00 (1.56-2.57)	1.42 (1.06-1.90)
Low wealth vs middle or high wealth	1.58 (1.25-2.00)	1.28 (0.99-1.64)	2.73 (2.06-3.61)	0.95 (0.69-1.30)
<High school degree vs ≥high school degree	1.54 (1.27-1.87)	1.31 (1.05-1.62)	0.99 (0.77-1.28)	1.20 (0.90-1.61)
Female vs Male	0.95 (0.79-1.15)	0.98 (0.79-1.22)	0.90 (0.70-1.16)	1.14 (0.85-1.52)

© 2025 Fayyad R et al. *JAMA Network Open*.

References:

1. Little RJA. Survey Nonresponse Adjustments for Estimates of Means. *International Statistical Review / Revue Internationale de Statistique*. 1986;*54*(2):139–157. doi:10.2307/1403140

© 2025 Fayyad R et al. *JAMA Network Open*.
