# Supplementary material for: Social Determinants of Self-Reported Health in Vulnerable Populations During a Polycrisis in Lebanon
Source: JAMA Netw Open. 2025 Oct 8;8(10):e2529733. doi: 10.1001/jamanetworkopen.2025.29733 (PMC12509029; doi:10.1001/jamanetworkopen.2025.29733)
Supplement: Supplement 2. — Community Action for Equity in Pandemic Preparedness and Control (CAEP) Study Group [file jamanetwopen-e2529733-s002.pdf]

Supplemental Online Content: Nonauthor Collaborators
*First name, last name, and suffix (if applicable) are required and will appear in PubMed.

*Group Name(s): Community Action for Equity in Pandemic Preparedness and Control (CAEP) Study Group

***First Name and Middle Initial(s)**	***Last Name**	***Suffix (eg, Jr, III)**	Academic Degrees	Institution	Location (city, state/province, country)	Role or Contribution, eg, chair, principal investigator	Group (if more than 1 Group listed in the byline) and/or Subgroup (eg, Steering Committee)
Nada	Melhem		PhD	American University of Beirut			CAEP Study Group
Fadi	El-Jardali		PhD	American University of Beirut			CAEP Study Group
Jocelyn	DeJong		PhD	American University of Beirut			CAEP Study Group
Aline	Germani		MPH	American University of Beirut			CAEP Study Group
