# Supplementary material for: Social Determinants of Self-Reported Health in Vulnerable Populations During a Polycrisis in Lebanon
Source: JAMA Netw Open. 2025 Oct 8;8(10):e2529733. doi: 10.1001/jamanetworkopen.2025.29733 (PMC12509029; doi:10.1001/jamanetworkopen.2025.29733)
Supplement: Supplement 3. — Data Sharing Statement [file jamanetwopen-e2529733-s003.pdf]

Data Sharing Statement

Fayyad. Social Determinants of Self-Reported Health in Vulnerable Populations During a Polycrisis in Lebanon. *JAMA Netw Open*. Published October 08, 2025. doi:10.1001/jamanetworkopen.2025.29733

Data
Data available: No

Additional Information
Explanation for why data not available: De-identified participant data can be obtained upon reasonable request from the Center for Research on Population and Health at the American University of Beirut (crph@aub.edu.lb).
